# Curcumin and Resveratrol Improve Muscle Function and Structure through Attenuation of Proteolytic Markers in Experimental Cancer-Induced Cachexia

**DOI:** 10.3390/molecules26164904

**Published:** 2021-08-13

**Authors:** Antonio Penedo-Vázquez, Xavier Duran, Javier Mateu, Adrián López-Postigo, Esther Barreiro

**Affiliations:** 1 Department-Muscle Wasting and Cachexia in Chronic Respiratory Diseases and Lung Cancer Research Group, IMIM-Hospital del Mar, Parc de Salut Mar, Health and Experimental Sciences Department (CEXS), Universitat Pompeu Fabra (UPF), Barcelona Biomedical Research Park (PRBB), 08003 Barcelona, Spain; ac.penedo94@gmail.com (A.P.-V.); alopez2@imim.es (A.L.-P.); 2 Scientific and Technical Department, Hospital del Mar-IMIM, 08003 Barcelona, Spain; xduran@imim.es; 3 Department of Pharmacy, Hospital del Mar, Parc de Salut Mar, 08003 Barcelona, Spain; fmateu@parcdesalutmar.cat; 4 Centro de Investigación en Red de Enfermedades Respiratorias (CIBERES), Instituto de Salud Carlos III (ISCIII), 08003 Barcelona, Spain

**Keywords:** cancer-induced cachexia mouse model, curcumin, resveratrol, muscle function and structure, sirtuin-1, troponin I, muscle proteolysis, atrophy signaling pathways

## Abstract

Muscle wasting and cachexia are prominent comorbidities in cancer. Treatment with polyphenolic compounds may partly revert muscle wasting. We hypothesized that treatment with curcumin or resveratrol in cancer cachectic mice may improve muscle phenotype and total body weight through attenuation of several proteolytic and signaling mechanisms in limb muscles. In gastrocnemius and soleus muscles of cancer cachectic mice (LP07 adenocarcinoma cells, N = 10/group): (1) LC-induced cachexia, (2) LC-cachexia+curcumin, and (3) LC-cachexia + resveratrol, muscle structure and damage (including blood troponin I), sirtuin-1, proteolytic markers, and signaling pathways (NF-κB and FoxO3) were explored (immunohistochemistry and immunoblotting). Compared to nontreated cachectic mice, in LC-cachexia + curcumin and LC-cachexia + resveratrol groups, body and muscle weights (gastrocnemius), limb muscle strength, muscle damage, and myofiber cross-sectional area improved, and in both muscles, sirtuin-1 increased, while proteolysis (troponin I), proteolytic markers, and signaling pathways were attenuated. Curcumin and resveratrol elicited beneficial effects on fast- and slow-twitch limb muscle phenotypes in cachectic mice through sirtuin-1 activation, attenuation of atrophy signaling pathways, and proteolysis in cancer cachectic mice. These findings have future therapeutic implications as these natural compounds, separately or in combination, may be used in clinical settings of muscle mass loss and dysfunction including cancer cachexia.

## 1. Introduction

Cachexia and muscle wasting are major systemic manifestations in many chronic diseases and in cancer [1,2]. Specifically, cancer-induced cachexia deteriorates the quality of life of the patients independently of the underlying tumor status [1,2]. The prognosis of the cancer patients is also severely influenced by the presence of cachexia and muscle wasting [1,2]. The etiology of cancer cachexia is complex, and several factors are involved.

Oxidative stress, inflammation, signaling pathways, and increased proteolysis are important contributors to the process of muscle wasting and cachexia, as has been shown in different studies [3,4,5,6,7]. For instance, in several models [3,4,5,6,7] a rise in proteolysis, proteolytic markers, and the expression of atrophy signaling pathways was shown in the diaphragm and limb muscles of tumor-bearing mice that developed severe cachexia. In the peripheral muscles of patients with oncologic cachexia, mechanisms that enhance proteolysis and lead to the loss of muscle mass and function were also shown along with increased oxidative stress and systemic inflammation [8]. Despite recent progress in the identification of the underlying biology leading to muscle wasting and cachexia, more therapeutic opportunities are still needed.

Polyphenols are a large family of phytochemicals with diverse chemical properties usually present in several plants, food, nutraceuticals, and species. Despite the reported beneficial effects in many investigations, the potential value of polyphenols as predictors of disease progression and staging may be questionable given their complex and variable structure and their interactions with other bioactive components from the diet [9]. The polyphenolic compound curcumin is the component of the turmeric plant (root of the *Curcuma longa* plant) with the largest reported effects on health. Important benefits of treatment with curcumin have been demonstrated on tissues through the action of a wide range of mechanisms. For instance, smooth muscle and endothelial cell senescence were hindered in response to curcumin therapy, as a result of sirtuin-1 activity [10]. Interestingly, the inhibition of NF-κB activity elicited by curcumin was shown to improve the phenotype and function of skeletal muscles in several models characterized by alterations of this tissue [11,12,13]. Furthermore, treatment of mice with the NF-κB inhibitor curcumin also favored the process of muscle regeneration in experimental models of disuse muscle atrophy [14,15]. In a subacute model of oncologic cachexia in rats, however, curcumin significantly reduced tumor growth, while it was not able to attenuate muscle protein loss [16]. Whether curcumin may exert anticachectic effects in other models of cancer cachexia of longer duration remains to be fully identified. 

Resveratrol is also a natural polyphenol extracted from grapes, red wine, peanuts, and other plants. It is a powerful antioxidant [17], while it also induced beneficial effects on several tissues probably through the action of sirtuin-1 activity [18,19,20,21]. As such, exercise and resveratrol through a sirtuin-1 dependent mechanism was shown to improve muscle biogenesis in mice [22]. Resveratrol also ameliorated the lifespan of animals as a result of different mechanisms [19,21,23]. Muscle injury also diminished in the gastrocnemius of the rats that received treatment with resveratrol [24]. In mice exposed to hindlimb immobilization, muscle recovery and regeneration following atrophy were also significantly favored by the action of resveratrol treatment [15]. In subacute in vivo models of cancer cachexia, however, resveratrol did not elicit an improvement in muscle wasting [25]. 

Whether beneficial effects on muscle mass loss and enhanced proteolysis may be seen in other models of cancer, cachexia needs to be identified. Selection of the best polyphenol with therapeutic purposes to be administered to animal models or patients is not simple due to the lack of reliable quantitative biomarkers [26]. In the current investigation, curcumin and resveratrol were selectively used as they are easily available and previous studies have proven their beneficial effects on skeletal muscles in diverse experimental models [15,26,27].

Hence, we hypothesized that treatment of muscle wasting with either curcumin or resveratrol in cancer-induced cachexia may attenuate the loss of muscle mass and function, muscle phenotype, and total body weight through mitigation of several proteolytic and signaling mechanisms in limb muscles of mice. Hence, the study objectives were that in two hindlimb muscles, namely gastrocnemius and soleus, of lung cancer (LC) cachectic mice treated with either curcumin or resveratrol for 15 consecutive days: (1) limb muscle strength and weight, (2) muscle fiber type and morphometry, (3) muscle structural abnormalities, (4) sirtuin-1 and muscle-specific protein content, (5) proteolytic markers including troponin I, and (6) signaling mechanisms were examined. A group of cancer cachectic mice that received no treatment with any of the polyphenolic compounds was used as the control animals. Female mice were used for the sake of consistency with previously reported results in our group [3,7,15,27,28,29]. Specifically, trans-resveratrol was used in the current investigation. 

## 2. Methods

### 2.1. Animal Experiments

#### 2.1.1. Tumor Inoculation and Treatments

LP07 cell line was acquired from an in vitro subculture of P07 LC tumor, which is a transplantable adenocarcinoma that naturally emerged in the lungs of a BALB/c mouse [3,29,30,31]. The cell line was validated for its characteristics as an identical tumor developer, tumor incidence, pathological features, and the potential to induce cachexia, like its progenitor P07 tumor [3,29,30,31]. The model used in the current study has been extensively validated [3,7,28,31]. Resveratrol was purchased from Fagron Ibérica (Terrassa, Barcelona, Spain): molecular weight 228.2 g, C_14_H_12_O_3_, 98.6% purity, and the trans conformation was used. Curcumin (CURCUMA COMPLEX 10,000 mg) was purchased from Airbiotic Health and Wellness Research Ltd. (Northumberland, UK), containing 95% purity.

#### 2.1.2. Experimental Protocol

Ten-week-old female BALB/c mice (weight ~20 g) were acquired from Harlan Interfauna Iberica SL (Barcelona, Spain). Female mice were used for practical reasons, since most investigations have also been carried out in this type of rodent in our group [3,7,15,27,28,29].

With the aim to reproduce a model of cancer cachexia, the mice were subcutaneously inoculated in the left flank (day 1) with the LP07 cells (4 × 10^5^), which were resuspended in 0.2 mL minimal essential medium (MEM), [3,29,30,31]. The research was conducted for a period of one month for all the groups (n = 10/group).

**Figure 1:**
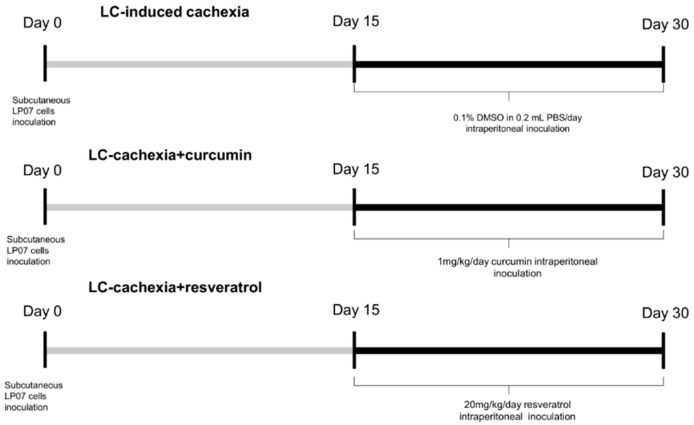
Schematic representation of the study protocol for all the groups of mice.

The study protocol is shown in Figure 1. Mice were subdivided randomly into three different groups: (1) LC control, inoculation of LP07 cells and concomitant daily intraperitoneal injection with saline for the last 15 days (LC-induced cachexia), (2) LC cachexia group treated with curcumin, inoculation of LP07 cells and concomitant daily intraperitoneal treatment with 1 mg/kg/day curcumin for the last 15 days (LC-cachexia+curcumin), and (3) LC cachexia mice treated with resveratrol, inoculation of LP07 cells and concomitant intraperitoneal treatment with 20 mg/kg/day resveratrol for the last 15 days (LC-cachexia + resveratrol, Figure 1). In this protocol, administration of the different treatments started on day 15, when the tumors were visible in the mice. Before that timepoint, administration of the treatments was not justified for ethical reasons as advised by the Animal Research Committee (see below). Moreover, also for ethical reasons, a nontumor control group of mice was not included in the investigation. Differences between nontumor animals and tumor-bearing mice have been extensively described in this model of lung cancer-induced cachexia [3,7,28,31].

Animals were kept under pathogen-free conditions with a 12:12 hour light–dark cycle. All the animal experiments were conducted in the animal facilities of the building Barcelona Biomedical Research Park (PRBB). Ethical regulations on animal experimentation set by the European Community Directive 2010/63/EU, Spanish legislation (*Real Decreto* 53/2013, BOE 34/11370-11421), and the European Convention for the Protection of Vertebrate Animals Used for Experimental and Other Scientific Purposes (1986) were all followed in the present study. All animal experiments were approved by the Animal Research Committee at PRBB (Animal Welfare Department in Catalonia, Spain, protocol # EBP-17-0005).

#### 2.1.3. Studies in Mice: In Vivo Measurements

The parameters food intake and total body weight were recorded on daily basis. Water and food were supplied ad libitum daily. In all the animals, grip strength was quantified using a grip strength meter (Bioseb, Vitrolles, France). Plasma samples were extracted on day 0, on day 15 before starting the different treatments, and on day 30, right before the sacrifice of the mice [3,15,27,28,29,31]. Limb strength and total body weight gains were calculated as the percentage of the measurements obtained on day 30 with respect to baseline measurements (day 0) [3,15,27,28,29,31].

#### 2.1.4. Sacrifice and Sample Collection

All the mice were sacrificed 30 days after the start of the study protocol. All the animals received an intraperitoneal injection containing 0.1 mL sodium pentobarbital (60 mg/Kg). Total anesthetic depth (pedal and blink reflexes) was verified in all the animals prior to their sacrifice. Animals were sacrificed upon diaphragm removal. Immediately afterwards, the gastrocnemius and soleus muscles were extracted from all the animals simultaneous to the time of sacrifice in order to keep the muscles oxygenated. A fragment of the muscle samples was snap-frozen in liquid nitrogen to be stored frozen at −80 °C up until further use. An additional part of the gastrocnemius and soleus muscles was paraffin-embedded for morphometrical analyses [3,15,27,28,29,31].

### 2.2. Biological Analyses

#### 2.2.1. Immunoblotting of 1D Electrophoresis

Immunoblotting was used to detect levels of the different antigens as also reported in previous investigations [3,15,27,28,29,31]. Stored frozen samples from the gastrocnemius and soleus muscles were homogenized in a buffer containing the following compounds: 50 mM 4-(2-hydroxyethyl)-1-piperazineethanesulfonic acid (HEPES), 150 mM NaCl, 100 mM NaF, 10 mM Na pyrophosphate, 5 mM ethylenediaminetetraacetic acid (EDTA), 0.5% Triton-X, 2 micrograms/mL leupeptin, 100 micrograms/mL phenylmethanesulfonyl fluoride (PMSF), 2 micrograms/mL aprotinin, and 10 micrograms/mL pepstatin A. The myofibrillar compartment was also isolated to detect the contractile proteins actin and myosin heavy chain (MyHC) as previously reported [3,15,27,28,29,31].

Proteins in each muscle sample were separated through electrophoresis, transferred to polyvinylidene difluoride (PVDF) membranes, and blocked with bovine serum albumin (BSA) to be incubated overnight with specific primary antibodies. The following primary antibodies were used in the investigation to detect the target antigens: NAD-dependent protein deacetylase sirtuin-1 (anti-sirtuin-1 antibody, ProteinTech Group Inc., Rosemont, IL, USA), nuclear factor kappa-light-chain-enhancer of activated B cells (NF-κB) p50 (anti-p50 antibody, Santa Cruz Biotechnology), Forkhead box O3 (FoxO-3) (anti-FoxO-3 antibody, Acris, Herford, Germany), MyHC (anti-MyHC antibody, clone A4.1025, Upstate-Millipore, Temecula, CA, USA), α-actin (anti-alpha-sarcomeric actin antibody, clone 5C5, Sigma-Aldrich, St. Louis, MO, USA) total ubiquitinated proteins (anti-protein ubiquitination antibody, Boston Biochem, Cambridge, MA, USA), 20S proteasome subunit C8 (anti-C8 antibody, Biomol, Plymouth Meeting, PA, USA), ubiquitin-ligase atrogin-1 (anti-atrogin-1 antibody, Acris), ubiquitin-ligase muscle ring finger (MURF)-1 (anti-MURF-1 antibody, Santa Cruz Biotechnology), and glyceraldehyde-3-phosphate dehydrogenase (GAPDH, anti-GAPDH antibody, Santa Cruz Biotechnology). 

Following an overnight incubation with the primary antibodies, horseradish peroxidase (HRP)-conjugated secondary antibodies (IgG) were also incubated for two more hours at room temperature. A chemiluminescence kit was also used to detect the specific antigen bands in the different immunoblots. 

Samples from the different groups were always run together for all the immunoblots. Moreover, membranes from all the samples were always analyzed simultaneously under identical exposure times for the sake of comparisons. Antibody specificity was tested by omission of the primary antibodies for each specific antigen. PVDF membranes were scanned using the spectral fluorescence imaging system Alliance Q9 Advanced (UVITEC, Cambridge, UK) using the software NineAlliance Q9 (UVITEC). Optical densities of the target protein bands were also calculated using the software NineAlliance Q9 (UVITEC). Optical densities obtained in each specific group of muscle and mice corresponded to those of the mean values of the different samples for each target antigen. The glycolytic enzyme GAPDH was used as the protein loading control in all the immunoblots.

#### 2.2.2. Enzyme-Linked ImmunoSorbent Assay (ELISA) Plasma Skeletal Muscle Troponin-I Levels

In the plasma compartment of the study animals, muscle troponin-I levels were detected using a specific ELISA kit (Troponin I ELISA kit, Elabscience Biotechnology Inc., Houston, Texas, USA) and standard procedures in our group [15,27,28]. Plasma samples and reagents were equilibrated to room temperature prior to the initiation of the assay. A standard curve (100 µL/standard) was always run with each assay following the manufacturer’s instructions. Samples were diluted (1:3 dilution) and equal volumes of each sample (100 µL) were loaded onto the plates. All the samples were incubated with 100 µL of biotinylated detection antibody at 37 °C for one hour. Following three washes with wash buffer, samples were additionally incubated with 100 µL HRP-secondary antibody at 37 °C for 30 min. The samples were washed five more times with wash buffer and were then incubated with substrate reagent at 37 °C for 15 minutes. The reaction was stopped using 50 µL of the stop solution. Optical densities were measured at 450 nm wavelength in a microplate reader (Infinite M200, TECAN, Männedorf, Switzerland). Intra-assay coefficients of variation for the measurements of plasma skeletal muscle troponin-I levels ranged from 2 to 10%. As all the samples were analyzed on the same day, no interassay coefficients of variation could be calculated.

#### 2.2.3. Muscle Fiber Typing and Morphometry

Paraffin-embedded gastrocnemius and soleus muscle sections were cut on a microtome (Leica RM 2035, Leica Biosystems, Nussloch, Germany) into three-micrometer sections. Slow- and fast-twitch muscle fibers were identified using immunohistochemical procedures with mouse specific monoclonal anti-MyHC I antibody (ab11083, Abcam) and anti-MyHC II antibody (ab51263, Abcam), respectively [15,27,28]. The cross-sectional area, mean least diameter, and proportions of type I and type II fibers were assessed using a conventional optical microscope (×20 objective, Olympus BX61, Olympus, Tokyo, Japan) coupled with an image-digitizing camera (Olympus U-TV1X-2, Olympus, Tokyo, Japan) and the Image J software (National Institute of Health, available at http://rsb.info.nih.gov/ij/, accessed on 1 April 2020). In each muscle cross-section, a minimum number of 100 fibers were counted for each limb muscle (gastrocnemius and soleus) in all the study groups of mice [15,27,28]. 

#### 2.2.4. Muscle Morphological Features

Three-micrometer paraffin-embedded sections of gastrocnemius and soleus of all groups of mice were used to assess the area fraction of normal and abnormal muscle. The sections were stained using hematoxylin-eosin to quantify the proportions of morphological features in the study muscles. Quantitative analyses were conducted using computer-assisted morphometric procedures. The setup consisted of an IBM-compatible computer with a stereology software package (The Gridder; WillRich Technologies, American Megatrends Inc., Georgia, United States) and a Nikon light microscope with a camera lucida. Using this software program, a grid consisting of 63 point-intercepts (7 × 9 rectangular pattern) was projected from the computer monitor via the camera lucida and superimposed onto the image of the muscle cross sections viewed down the light microscope. The observer was blinded to the identity of all the slides. The following features were evaluated in each one of the 63 point-intercepts of every image: (1) normal muscle; (2) internal nuclei; (3) inflammatory cells; (4) lipofuscin; (5) abnormal fibers; (6) necrotic or inflamed fibers; (7) blood vessels. The sections were divided into normal muscle (1), abnormal muscle (2–6), or blood vessels (7), and the percentages of normal and abnormal muscle were calculated for each sample type [4].

#### 2.2.5. Statistical Analysis

Results are presented as mean values and standard deviations. The normality of the study variables was explored using the Shapiro–Wilks test. Sample size was calculated according to body weight change in all the study groups. Ten mice in each group were sufficient to achieve a 90% statistical power in the investigation. 

The variables of food intake and percentage of change of both total body weight and limb strength for all the study mouse groups are represented in a table, while the biological variables are represented in figures (whiskers and box plots).

Potential differences among the study groups were explored using one-way analysis of variance (ANOVA) with Dunnett’s post hoc analysis (to adjust for multiple comparisons among the study groups) for all the study variables. A level of significance of *p* ≤ 0.05 was established. All the statistical analyses were performed using the software for Statistics and Data Science (STATA, StataCorp LLC, College Station, TX, USA).

## 3. Results

### 3.1. Physiological Characteristics of the Study Animals

Compared to nontreated cachectic mice, the loss of body weight was significantly attenuated in the cachexia groups treated with either curcumin or resveratrol, while food intake did not vary across groups (Table 1). The weight of the gastrocnemius and soleus muscles significantly improved in the cachexia groups treated with either curcumin or resveratrol compared to nontreated cachectic mice (Table 1). In comparison with the nontreated cachectic mice, tumor weight significantly decreased in the cachectic animals treated with resveratrol (≈30% reduction), whereas no significant differences were seen in the mice treated with curcumin (17% reduction, Table 1). Limb strength gain was greater in the animals treated with either curcumin (larger increase) or resveratrol compared to the nontreated cachectic mice (Table 1).

### 3.2. Structural Phenotypic Characteristics

**Figure 2:**
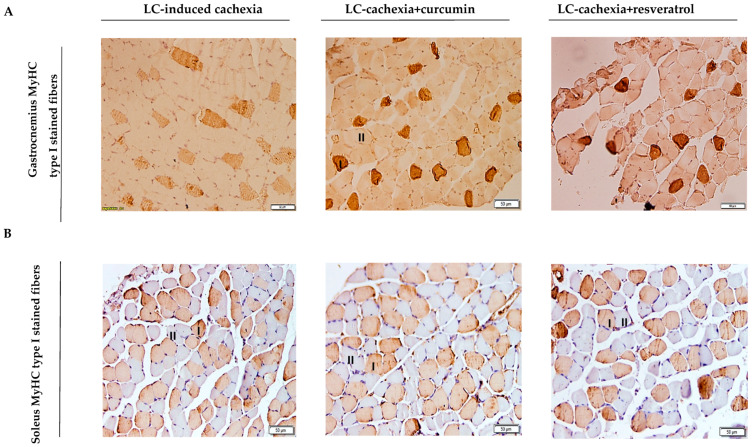
(**A**) Representative images of muscle fibers (×200) of gastrocnemius (**A**) and soleus (**B**) muscle of all study groups of mice. Slow-twitch fibers (I symbol within the fibers) were positively stained with the corresponding antibody (brown color), while the nonstained fibers were fast-twitch ones (II symbol within the fibers).

**Figure 3:**
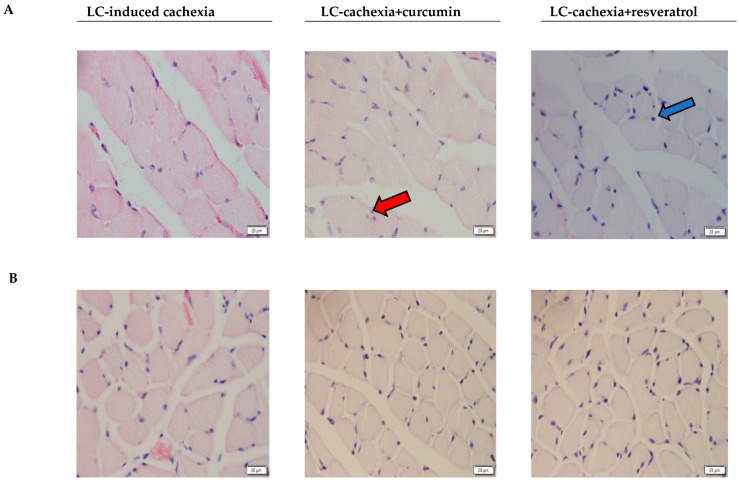
Representative images of cross gastrocnemius (**A**) and soleus (**B**) muscle sections with H&E staining. An example of an internal nucleus (red arrow) and an inflammatory cell (blue arrow) are indicated.

No significant improvements in the proportions of muscle fiber types were detected in any of the analyzed muscles in the mice treated with the polyphenolic compounds (Table 2 and Figure 2A,B). Importantly, the areas of both type I and type II fibers were significantly greater in the gastrocnemius and soleus muscles of cachectic mice treated with either curcumin or resveratrol than in nontreated cachectic rodents (Table 2 and Figure 2A,B). The proportions of muscle abnormalities, including the proportions of internal nuclei and inflammatory cells, were significantly lower in the gastrocnemius and soleus of the cachectic mice treated with either curcumin or resveratrol compared to nontreated cachectic animals (Table 2 and Figure 3A,B).

### 3.3. Sirtuin-1 Protein Content

**Figure 4:**
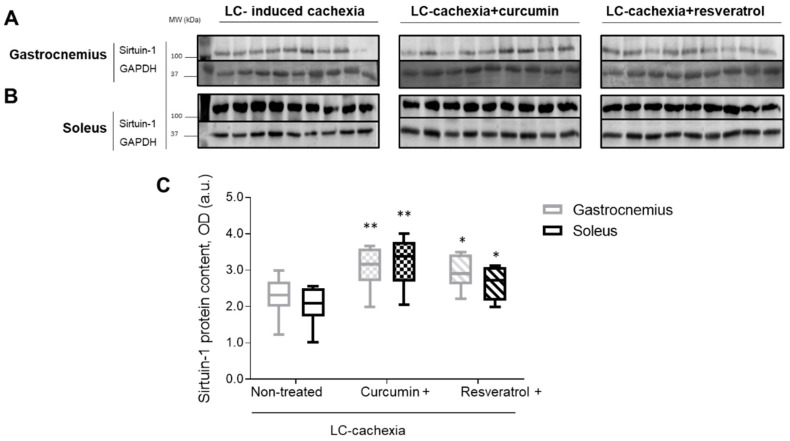
Representative immunoblots of sirtuin-1 and GAPDH proteins in the gastrocnemius (**A**) and soleus (**B**) muscles of all study groups of mice. Definition of abbreviations: MW, molecular weight; kDa, kilodalton; OD, optical densities; a.u., arbitrary units; LC, lung cancer; GAPDH, glyceraldehyde-3-phosphate dehydrogenase; (**C**) box plots of sirtuin-1 protein content in gastrocnemius and soleus muscles of the different study groups of mice, as measured by optical densities in arbitrary units (OD, a.u.). Statistical significance is represented as follows: *, *p* ≤ 0.05; **, *p* ≤ 0.01 between any of the study groups and the LC-induced cachexia mice in gastrocnemius and soleus muscles.

Sirtuin-1 protein levels significantly increased in both limb muscles of the cachectic mice treated with either curcumin or resveratrol compared to nontreated cachectic rodents (Figure 4A–C). 

### 3.4. Muscle Specific Proteins

**Figure 5:**
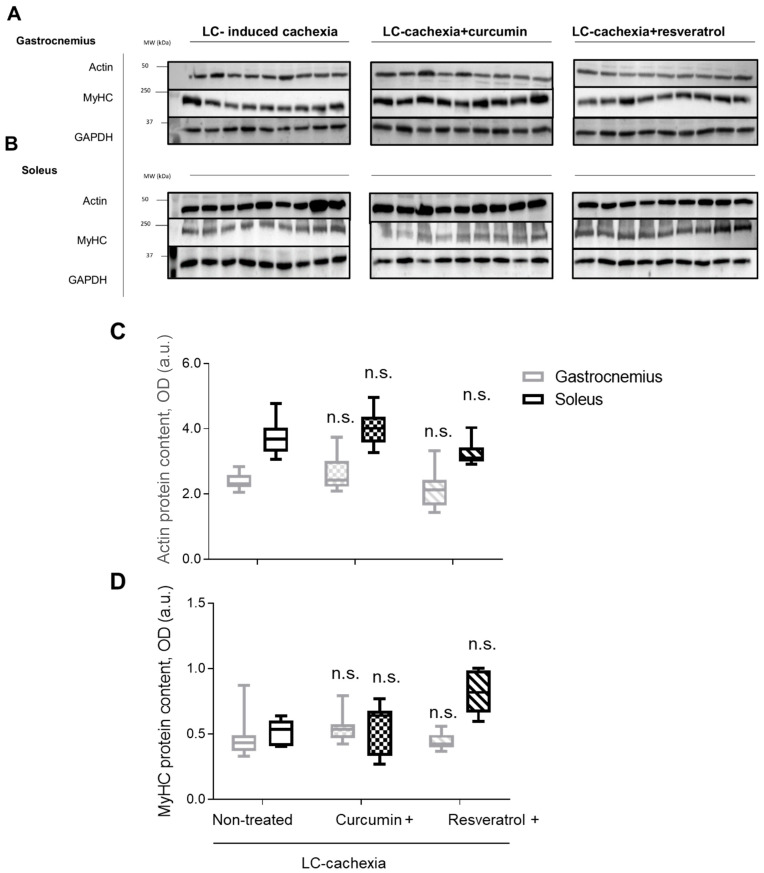
Representative immunoblots of actin, MyHC, and GAPDH protein content in the gastrocnemius (**A**) and soleus (**B**) muscles of all study groups of mice. Box plots of actin (**C**) and MyHC (**D**) protein content in gastrocnemius and soleus muscles of the different study groups of mice, as measured by optical densities in arbitrary units (OD, a.u.). Definition of abbreviations: a.u., arbitrary units; MW, molecular weight; kDa, kilodalton; LC, lung cancer; MyHC, myosin heavy chain; GAPDH, glyceraldehyde-3-phosphate dehydrogenase. ns, non significant.

No significant differences in protein levels of actin or myosin heavy chain were detected in either gastrocnemius or soleus muscles among the study groups of mice (Figure 5A–D).

### 3.5. Muscle Proteolytic Markers

**Figure 6:**
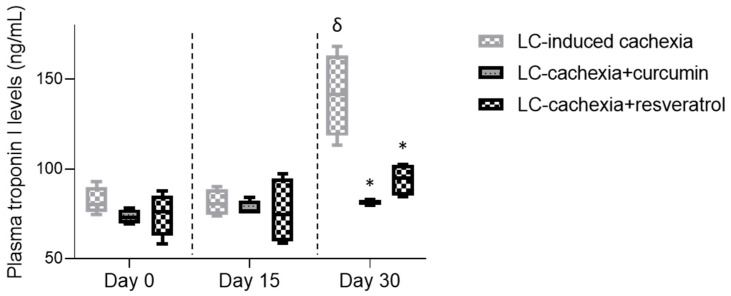
Box plots of plasma troponin I (ng/mL) of the different study groups of mice on days 0, 15 and 30. Definition of abbreviations: ng, nanogram; mL, milliliter; LC, lung cancer. Statistical significance is represented as follows: δ, *p* ≤ 0.05 levels in plasma of LC-induced cachexia mice between day 30 and day 0: *, *p* ≤ 0.05; plasma levels in cachectic mice treated with either curcumin or resveratrol and the nontreated cachectic mice at day 30.

**Figure 7:**
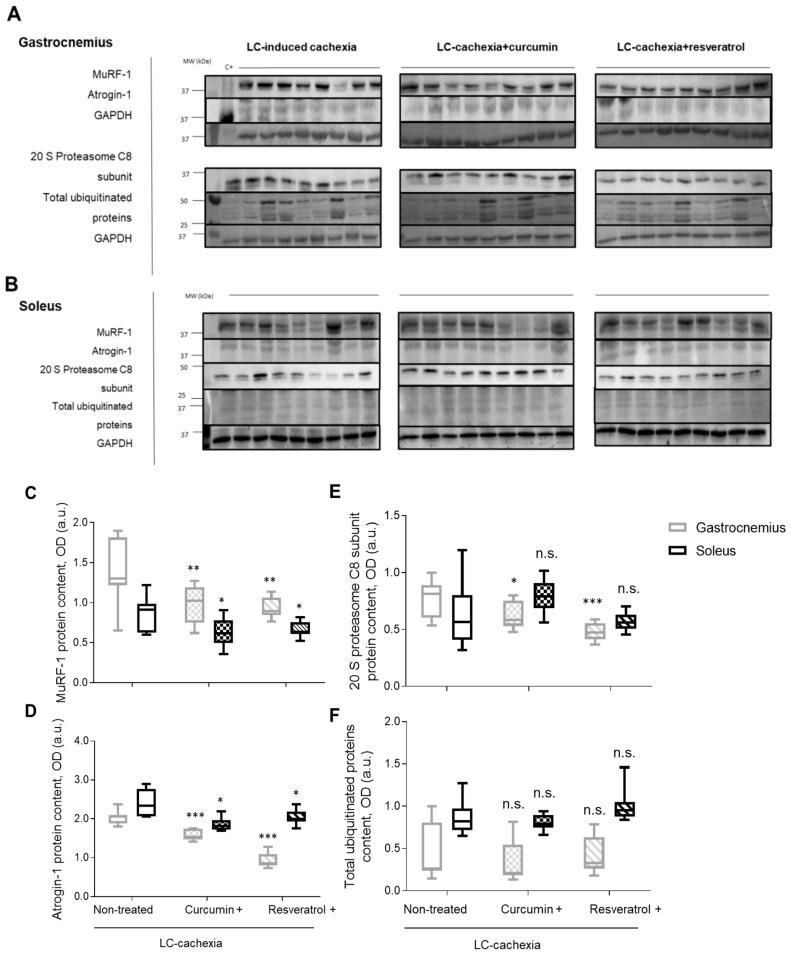
Representative immunoblots of C8-20S, total ubiquitinated proteins, MuRF-1, Atrogin-1, and GAPDH protein content in the gastrocnemius (**A**) and soleus (**B**) muscles of all study groups of mice. Box plots of MuRF-1 (**C**), Atrogin-1 (**D**), 20S subunit of the C8 proteasome marker (**E**), and total ubiquitinated proteins (**F**) protein content in gastrocnemius and soleus muscles of the different study groups of mice, as measured by optical densities in arbitrary units (OD, a.u.). (Definition of abbreviations: OD, optical densities; a.u., arbitrary; MW, molecular weight; kDa, kilodalton; LC, lung cancer; C8-20S, 20S proteasome alpha subunit; MuRF-1, muscle ring finger protein 1; GAPDH, glyceraldehyde-3-phosphate dehydrogenase. Statistical significance is represented as follows: *, *p* ≤ 0.05; **, *p* ≤ 0.01; ***, *p* ≤ 0.001, ns, non significant, between any of the study groups and the LC-induced cachexia mice in gastrocnemius and soleus muscles.

A significant increase in plasma troponin I levels was detected in the cachectic mice only on day 30 compared to levels on days 0 and 15 (Figure 6). Interestingly, a significant decline in systemic troponin I levels was seen in mice treated with either curcumin or resveratrol only in the 30-day time-point (Figure 6). A significant reduction in protein levels of MuRF-1 and atrogin-1 was observed in the gastrocnemius and soleus muscles of cachectic mice treated with either curcumin or resveratrol compared to limb muscles in the nontreated mice (Figure 7A–D). Protein levels of the 20S subunit of the C8 proteasome marker were significantly lower only in the gastrocnemius, but not in the soleus, of the cachectic mice treated with either curcumin or resveratrol compared to nontreated cachectic animals (Figure 7A,B,E). Total levels of protein ubiquitination did not significantly differ among the study groups for any of the study muscles (Figure 7A,B,F).

### 3.6. Muscle Atrophy Signaling Markers

**Figure 8:**
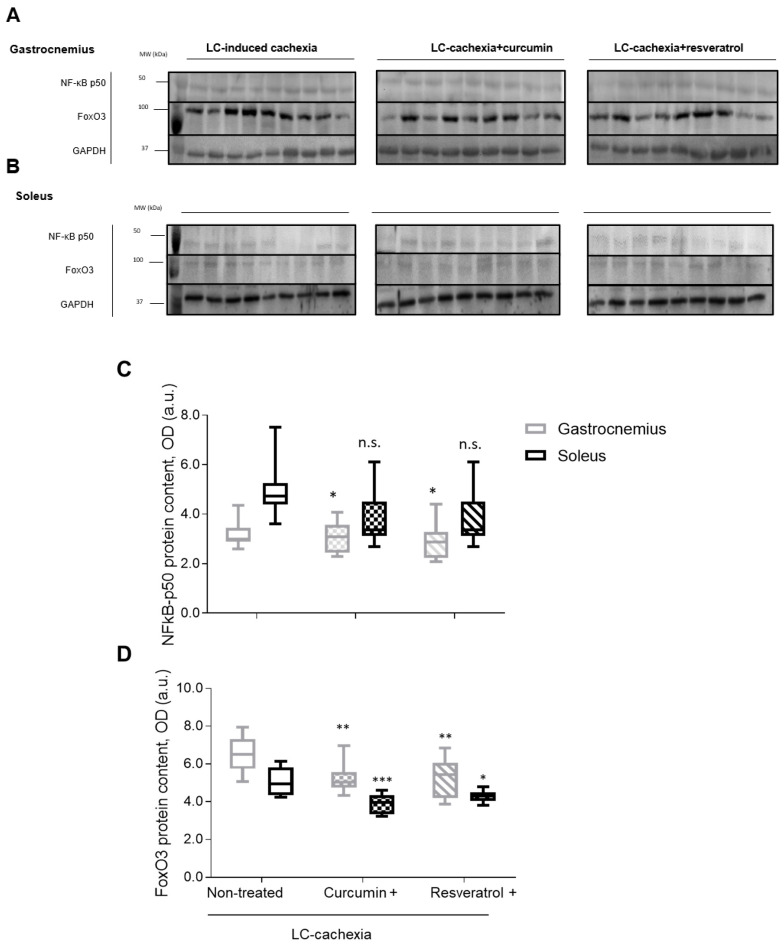
Representative immunoblots of NF-κB p50, FoxO3, and GAPDH protein content in the gastrocnemius (**A**) and soleus (**B**) muscles of all study groups of mice. Box plots of Nf-κB p50 (**C**) and FoxO3 (**D**) protein content in gastrocnemius and soleus muscles of the different study groups of mice, as measured by optical densities in arbitrary units (OD, a.u.). Definition of abbreviations: OD, optical densities; a.u., arbitrary units; MW, molecular weight; kDa, kilodalton; LC, lung cancer; NF-κB, nuclear factor kappa-light-chain-enhancer of activated B cells; FoxO3, Forkhead box O3; GAPDH, glyceraldehyde-3-phosphate dehydrogenase. Statistical significance is represented as follows: *, *p* ≤ 0.05; **, *p* ≤ 0.01; ***, *p* ≤ 0.001, ns, non significant, between any of the study groups of mice treated with either curcumin or resveratrol and the LC-induced cachexia mice in gastrocnemius and soleus muscles.

Total levels of the signaling marker NF-κB p50 significantly decreased in the soleus, but not in the gastrocnemius, of the cachectic mice treated with either curcumin or resveratrol compared to nontreated animals (Figure 8A–C). Total protein levels of FoxO3 significantly declined in both limb muscles of the cachectic mice treated with either curcumin or resveratrol compared to nontreated mice (Figure 8A,B,D). 

## 4. Discussion

In the current study, the most relevant results were that treatment with the polyphenolic compounds curcumin and resveratrol via a sirtuin-1 mechanism elicited beneficial effects in tumor-bearing mice with severe cachexia as demonstrated by the clear improvement seen in body and muscle weights as well as in limb muscle strength. Importantly, cross-sectional areas of both slow- and fast-twitch muscle fibers also significantly increased in the limb muscles of the cachectic mice treated with either curcumin or resveratrol. Moreover, muscle proteolysis and the expression of the target proteolytic markers was also attenuated by the effects of curcumin and resveratrol in both slow- and fast-twitch type muscles in this model of cancer cachexia. Expression levels of atrophy signaling markers were also attenuated as a result of treatment with either resveratrol or curcumin in both limb muscle types. The most relevant findings are discussed below. 

In this model of cancer cachexia, treatment with either resveratrol or curcumin elicited an improvement in muscle phenotype of the target limb muscles. Furthermore, the weights of the gastrocnemius and soleus muscles were significantly greater in the cachectic mice treated with the polyphenolic compounds. The degree of muscle structural abnormalities also declined in the hindlimb muscles of the treated animals. Altogether these are relevant findings that may partly account for the amelioration observed in limb muscle strength in the cachectic mice treated with the polyphenolic compounds compared to the nontreated animals. In line with this, similar results were also reported in different models, in which the mouse limb muscles were exposed to disuse muscle atrophy [15,27] and animals received treatment with curcumin and resveratrol. 

Biological events such as DNA repair, cell survival, and aging [32,33] are regulated by the nicotinamide adenine dinucleotide (NAD)+ dependent histone deacetylase sirtuin-1. Muscle proteolysis may also be modulated by the action of sirtuin-1 activity [27]. In keeping with this, a decline in sirtuin-1 protein levels was also demonstrated in limb muscles and myotubes of patients with COPD and severe muscle wasting [34], as well as in mice exposed to hindlimb unloading [15,27]. In the present investigation, treatment with resveratrol and curcumin of the cancer cachectic mice elicited favorable effects in a similar fashion in both slow- and fast-twitch limb muscle types. Interestingly, a significant rise in sirtuin-1 protein content was detected in the gastrocnemius and soleus muscles of mice treated with either curcumin or resveratrol. These relevant findings suggest that the benefits observed in the cachectic limb muscles are mediated to a great extent by the actions of sirtuin-1. Furthermore, skeletal muscle damage, a common feature in cancer-induced cachexia [3,4,7,28,31], also decreased in the animals treated with any of the two polyphenolic compounds compared to the nontreated mice. Interestingly, the reduction in muscle structural abnormalities was further confirmed by the significant reduction observed in plasma troponin I levels of the treated mice. In addition, protein levels of the E3 ligases MuRF-1 and atrogin-1 and the 20S proteasome C8 subunit also decreased in the limb muscles in response to treatment with either resveratrol or curcumin in the cachectic rodents. Collectively, these results suggest that the polyphenolic compounds most likely exerted their beneficial effects on muscle phenotype and function through the action of sirtuin-1, as also previously reported in models of disuse muscle atrophy [15,27]. In fact, curcumin was demonstrated to attenuate the expression of atrophy signaling pathways and muscle protein degradation [27]. 

Another point that warrants attention is to explore whether the administration of a combined therapy with curcumin and resveratrol may enhance the beneficial effects on the skeletal muscles in this mouse model of cancer-associated cachexia as previously shown to occur in patients with chronic kidney failure [35]. Indeed, this will be an excellent avenue for research in the near future. Another aspect that also deserves special attention was the significant reduction seen in tumor weight in the cachectic mice treated with resveratrol (30%), but not in those treated with curcumin (17%). Despite that the reduction in tumor size was not very large, in the animals treated with resveratrol, a potential direct effect on the muscles resulting from the smaller tumor burden cannot be ruled out. On the other hand, curcumin predominantly exerted its beneficial effects directly on muscle proteolysis and function. Indeed, the improvement in limb muscle strength elicited by curcumin was larger than that induced by resveratrol. Previous results put forward that resveratrol also significantly reduced tumor burden through inhibition of proliferation and induction of autophagy in mice with ovarian cancer for several weeks [36].

Resveratrol-related effects on health and lifespan are dependent on the activation of sirtuin-1 [37]. Muscle mass and function also significantly improved in elderly subjects in response to combined treatment with aerobic exercise training and resveratrol [38]. In a model of muscle-specific sirtuin-1-deficient mice, resveratrol elicited a beneficial effect on mitochondrial function and biogenesis [22]. On the other hand, sirtuin-1 has also been implicated in the negative regulation of insulin-like growth factor (IGF)-I and downstream Akt/mTOR signaling in skeletal muscles during senescence [39]. In one review [39], the role of sirtuin-1 in maintaining skeletal muscle mass in healthy aging was thoroughly discussed. Furthermore, in mice, resveratrol was also shown to induce beneficial effects on the process of muscle regeneration following unloading for several days [15].

Importantly, it has been recently proposed that resveratrol may exert dichotomic effects in different experimental models [40,41]. Previous work clearly demonstrated that tyrosyl-tRNA synthetase is a biological and physiological target of resveratrol and that this step is crucial in the conversion of trans- to cis-resveratrol [41]. Also, trans-resveratrol was shown to convert to cis-resveratrol in physiological processes, and cis- and trans-resveratrol forms were identified in the metabolic profiles of cells and tissues exposed to trans-resveratrol treatment [40,42]. Thus, the trans- to cis- conversion accounts for a novel thiol-dependent mechanism that mediates the favorable effects of resveratrol in in vivo models [40]. The tyrosyl-tRNA synthetase signaling mechanism probably accounts for most of the beneficial effects of resveratrol, including anti-inflammatory properties [43]. However, full elucidation of the biological significance of the two distinct conformations of resveratrol is still needed in different models. Future research should focus on the identification of whether cis- or trans- conformations exert the beneficial effects seen in different experimental models.

In the present investigation, expression of the signaling pathway NF-κB p50 subunit declined only in the gastrocnemius of the animals treated with the polyphenolic compounds compared to nontreated mice. These findings imply that the NF-κB pathway partly signaled the process of loss of muscle mass and function in the cachectic mice. Hence, it is likely that the decrease in NF-κB pathway expression observed in the mice treated with either resveratrol or curcumin partly elicited the beneficial effects seen on muscle function, structure, and biology. Likewise, similar effects were also reported in other models such as in aged muscles, in which NF-κB was also involved [19,20,21,23]. 

Importantly, expression levels of FoxO3 also experienced a decline in both muscle types as a result of the treatment with either resveratrol or curcumin of the cachectic mice. These relevant observations suggest that FoxO3 may be is a prominent pathway in this model of muscle wasting and that the polyphenolic compounds may also exert their beneficial effects through attenuation of FoxO3 signaling pathway. These results also confirm previous findings reported in other models of muscle wasting and sarcopenia, in which FoxO3 was also involved in the process of muscle wasting [8,28]. 

### Study Critique

A limitation in this study is related to the potential extrapolation of the results to clinical settings of patients with cancer-associated cachexia. In view of the present findings, future investigations targeted to identify whether resveratrol and curcumin or even other polyphenolic compounds may attenuate muscle proteolysis in patients with oncologic cachexia should be designed. On other hand, it should also be mentioned that previous investigations did not show positive effects on muscle wasting in rats or mice in subacute (7–14 days) cancer-induced cachexia models [16,25]. In the latter model [25], in vitro administration of resveratrol did elicit beneficial effects as a reduction in protein degradation as observed in rat limb muscles. The nature of the cancer cells inoculated to the rodents, the different conformations of resveratrol (cis- and trans-) [40], the different doses used in vivo and in vitro, and the duration of the experimental models may account for discrepancies or differences in the reported results among studies [16,25,40].

Despite these concerns, in the present study, a significant improvement in limb muscle strength was observed in the cachectic mice treated with either polyphenolic compound. In the mice treated with curcumin, this improvement was even greater, while tumor size did not differ significantly from the nontreated controls. Thus, curcumin exerted anticachectic effects in this mouse model of cancer cachexia.

Another potential limitation was related to the use of female mice in the study. For the sake of coherence and consistency with previously published results [3,7,15,27,28,29,31], female mice were used in the current investigation. Furthermore, cancer cachexia usually develops at a relatively late stage in life, time at which differences between men and female patients are less likely. 

## 5. Conclusions

The polyphenolic compounds curcumin and resveratrol elicited beneficial effects on fast- and slow-twitch limb muscle types in cachectic mice through attenuation of atrophy signaling pathways and induction of a sirtuin-1 dependent mechanism. Limb muscle strength, body and muscle weight, and the size of both slow- and fast-twitch myofibers along with reduced muscle damage and troponin I levels notably improved in response to treatment with either curcumin or resveratrol through attenuation of muscle proteolysis in cancer cachectic mice. Curcumin exerted more powerful anticachectic effects as tumor size was not significantly modified by this compound, while a larger improvement in muscle function, structure, and biology was demonstrated in the cancer cachectic mice. Future studies should specifically target the profile of the biological and physiological outcomes resulting from the two distinct resveratrol conformations. Furthermore, the potential beneficial enhancement of combination therapy with curcumin and resveratrol on cachectic muscles should also be explored in future investigations. The findings reported herein have future therapeutic implications, as these natural compounds may be used in clinical settings for muscle mass loss and dysfunction including cancer cachexia. 

## Figures and Tables

**Table 1:** Physiological parameters in the experimental groups of mice.

	LC-Induced Cachexia (N = 10)	LC-Cachexia + Curcumin (N = 10)	LC-Cachexia + Resveratrol (N = 10)
Age at baseline (weeks)	10	10	10
Body weight at baseline (g)	20.43 (1.08)	20.31 (1.13)	20.3 (1.08)
Final body weight (g)	15.76 (1.67)	18.5 (1.31) ***	19.25 (1.55) ***
Body weight gain (%)	−22.87 (6.98)	−8.87 (4.35) ***	−5.22 (3.8) ***
Food intake (g/24 h)	2.53 (0.52)	2.64 (0.58)	2.69 (0.58)
Gastrocnemius weight (g)	0.086 (0.01)	0.105 (0.01) ***	0.104 (0.008) ***
Soleus weight (g)	0.0064 (0.001)	0.0075 (0.001) *	0.0073 (0.001) *
Tumor weight (g)	2.136 (0.56)	1.77 (0.41) (17%)	1.49 (0.79) * (30%)
Limb strength gain (%)	−25.01 (2.29)	−1.51 (3.29) ***, +93%	−5.27 (6.9) ***, +78%

Variables are presented as mean (standard deviation). Abbreviations: LC, lung cancer; g, grams; h, hour. Statistical significance is represented as follows: * *p* ≤ 0.05; *** *p* ≤ 0.001 between any of the cachectic mice treated with either curcumin or resveratrol and the nontreated LC-induced cachexia mice.

**Table 2:** Structural characteristics of the gastrocnemius and soleus muscles in the study groups of mice.

	Muscle	LC-Induced Cachexia (N = 10)	LC-Cachexia + Curcumin (N = 10)	LC-Cachexia + Resveratrol (N = 10)
**Muscle fiber type, %**				
Type I fibers	Gastrocnemius	12.34 (1.9)	16.03 (4.4)	14.16 (3.4)
	Soleus	49.08 (5.01)	52.98 (9.38)	54.87 (5.94)
Type II fibers	Gastrocnemius	87.65 (1.9)	83.97 (4.4)	85.83 (3.4)
	Soleus	50.92 (5.01)	47.02 (9.38)	45.13 (5.94)
**Cross-sectional areas**				
Type I fibers (µm^2^)	Gastrocnemius	499.34 (71.9)	695.06 (136.63) *, +34%	711.44 (240.26) ***, +36%
	Soleus	528.65 (111.06)	799 (159.48) **, +51%	770.66 (102.86) **, +46%
Type II fibers (µm^2^)	Gastrocnemius	464.79 (53.88)	688.04 (132.61) ***, +49%	648.2 (134.2) **, +34%
	Soleus	443.16 (69.50)	603.17 (103.95) **, +36%	592.01 (99.27) *, +34%
**Muscle structural abnormalities, %**				
Total abnormal fraction	Gastrocnemius	3.8 (0.84)	2.85 (0.94) *	1.9 (0.27) ***
	Soleus	9.25 (1.88)	3.57 (0.55) ***	3.73 (0.39) ***
Internal nuclei	Gastrocnemius	2.34 (0.54)	2.23 (0.8)	1.21 (0.2) **
	Soleus	6.08 (1.36)	3.17 (0.43) ***	2.3 (0.5) ***
Inflammatory cells	Gastrocnemius	1.26 (0.59)	0.5 (0.32) *	0.58 (0.31) *
	Soleus	1.56 (1.48)	0.36 (0.29) *	0.49 (0.3) *

Variables are presented as mean (standard deviation). Abbreviations: LC, lung cancer; µm^2^, square micrometers. Statistical significance is represented as follows: *, *p* ≤ 0.05; **, *p* ≤ 0.01; ***, *p* ≤ 0.001 between any of the cachectic mice treated with either curcumin or resveratrol and the nontreated LC-induced cachexia mice.

## Data Availability

Not available.

## Sample Availability

Not available.
